# Serum Macro TSH Level is Associated with Sleep Quality in Patients with Cardiovascular Risks – HSCAA Study

**DOI:** 10.1038/srep44387

**Published:** 2017-03-13

**Authors:** Manabu Kadoya, Sachie Koyama, Akiko Morimoto, Akio Miyoshi, Miki Kakutani, Kae Hamamoto, Masafumi Kurajoh, Takuhito Shoji, Yuji Moriwaki, Masahiro Koshiba, Tetsuya Yamamoto, Masaaki Inaba, Mitsuyoshi Namba, Hidenori Koyama

**Affiliations:** 1 Department of Internal Medicine, Division of Diabetes, Endocrinology and Metabolism, Hyogo College of Medicine, 1-1 Mukogawa-cho, Nishinomiya, Hyogo 663-8501, Japan; 2 Department of Clinical Laboratory Medicine, Hyogo College of Medicine, 1-1 Mukogawa-cho, Nishinomiya, Hyogo 663-8501, Japan; 3 Department of Endocrinology, Metabolism and Molecular Medicine, Osaka City University Graduate School of Medicine, Osaka 545-8585, Japan

## Abstract

Macro thyroid-stimulating hormone (TSH) has been reported to be associated with seasonality and regulated by changes in day length in rodents, different from free TSH. In the present study, we investigated structural differences between macro TSH and free TSH levels in human serum, as well as the association of macro TSH with sleep quality. We enrolled 314 patients registered in the Hyogo Sleep Cardio-Autonomic Atherosclerosis (HSCAA) study. Sleep quality shown by actigraphy, sleep physical activity, and percent sleep in all and TSH closely matched subjects were significantly associated with high macro TSH levels. Macro and free TSH were similarly increased following thyrotropin-releasing hormone (TRH) stimulation, while circadian changes associated with those were distinct. To further analyze the structure of macro TSH, serum samples were separated by gel filtration chromatography. Although treatment with glycosidase did not affect morbidity, the macro TSH fraction had a markedly low affinity to the Con A column as compared with free TSH, indicating a distinct glycosylation structure. In conclusion, an increase in serum macro TSH is associated with low sleep quality and regulated in a manner distinct from free TSH, potentially due to an altered glycosylation structure.

Behavioral factors have been increasingly recognized in regard to development, prevention, and treatment of cardiovascular diseases (CVD)^1^,^2^. Among various behavioral factors, sleep conditions have been shown to be associated with CVD and mortality^3^,^4^, and we recently revealed potential mechanisms for these associations in reports of a cohort study [Hyogo Sleep Cardio-Autonomic Atherosclerosis (HSCAA) study]^5^,^6^.

Associations between thyroid function and sleep conditions have been found in patients with sleep apnea syndrome^7^. Thyroid-stimulating hormone (TSH; thyrotropin), a pituitary hormone that stimulates the thyroid gland to produce thyroid hormone^8^, has been shown to be elevated in patients with severe sleep apnea syndrome^7^. TSH synthesis in the anterior pituitary is stimulated by thyrotropin-releasing hormone (TRH) and inhibited by thyroid hormone in a classical endocrine negative-feedback loop. Thus, TSH level in serum is considered to be a sensitive marker for dysregulated thyroid function and targeted to treat related conditions. In hypothyroidism, an increase in body weight appears to be associated with sleep-disordered breathing through reduction in the basic metabolic rate, narrowing of the pharynx by myxedema, dysregulation of pharyngeal dilator muscles, and suppression of the respiratory center.

In patients with hypothyroidism, circulating TSH (total TSH) in serum is composed of free form (free TSH) and macro-molecular form (macro TSH). Macro TSH is a macromolecular complex of TSH and anti-TSH immunoglobulin. Of note in rodents, elevating macro TSH level was associated with seasonal physiology and behavior regulated by changes in day length^9^. Furthermore, macro TSH is secreted or complex-formed at the pars tuberalis (PT) adjacent to the pituitary. However, it is not known whether this type of macro TSH also exists in the circulation or is similarly regulated by sleep cycle in humans.

To address this question, we investigated the relationship between serum macro TSH and sleep quality, which was quantitatively determined by actigraphy findings, and also analyzed the structure of macro TSH in patients with cardiovascular risk factors.

## Results

**Figure 1:**
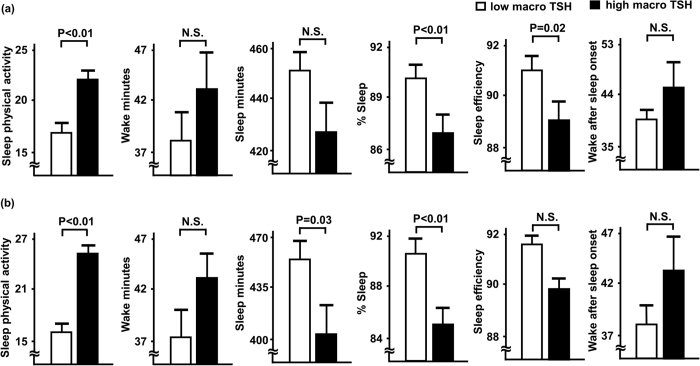
Comparisons of sleep parameters between patients with low and high macro TSH levels. Low and high macro TSH levels were determined based on the median value (77.6%). Comparisons of sleep parameters between the high and low macro TSH groups for all patients (**a**), and total TSH levels matched patients (**b**) are shown. Each column represents the mean ± standard error. Open columns: low macro TSH group; closed columns: high macro TSH group. TSH, thyroid stimulating hormone; N.S., not significant.

Table 1 shows comparisons of clinical characteristics between the high and low macro TSH groups. We also set the subgroups (Table 1, right column) with total TSH levels closely matched, to neglect the influence of subclinical hypothyroidism. In both total and subgroup analyses, body mass index, and prevalence of dyslipidemia and diabetes mellitus were significantly higher in the high macro TSH group. We also compared sleep parameters between the low and high macro TSH groups (Fig. 1). For all patients (Fig. 1a), sleep physical activity was significantly (P < 0.01) higher, while percent sleep (P < 0.01) and sleep efficiency (P = 0.02) were significantly lower in patients in the high macro TSH group. Wake and sleep minutes, and time awake after sleep onset were not significantly different between the groups. As for total TSH levels in closely matched patients (Fig. 1b), sleep physical activity (P < 0.01) was still significantly higher, while sleep minutes (P = 0.03) and percent sleep (P < 0.01) were significantly lower in patients with high macro TSH. On the other hand, wake minutes, sleep efficiency, and time awake after sleep onset were not significantly different between the subgroups. Table 2 shows results of multiple linear regression analysis of associations between high macro TSH and sleep parameters, which were fully adjusted for age, sex, and body mass index, as well as presence of hypertension, dyslipidemia, and diabetes mellitus. In all patients, even after such adjustments, high macro TSH remained significantly and independently associated with sleep physical activity (β = 0.145, P = 0.01) and percent sleep (β = −0.150, P < 0.01), but not with sleep efficiency. As for total TSH levels closely matched patients, high macro TSH was also significantly and independently associated with sleep physical activity (β = 0.288, P < 0.01) and percent sleep (β = −0.263, P < 0.01).

**Figure 2:**
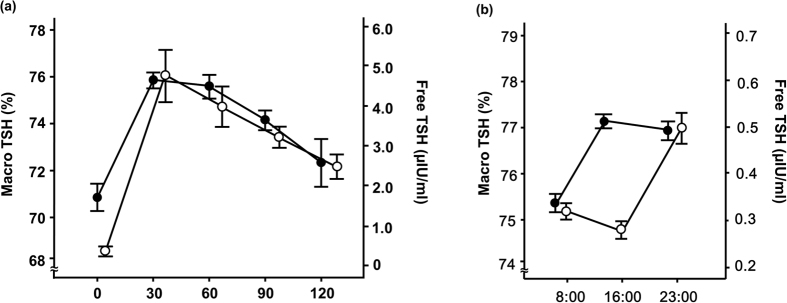
TRH stimulation test (**a**) and diurnal regulation (**b**) of macro TSH levels. Each plot represents the mean ± standard error (u = 5). Open circles, free TSH; closed circles, macro TSH. TSH, thyroid stimulating hormone; TRH, thyrotropin releasing hormone.

Results of TRH stimulation tests, and circadian changes in macro TSH and free TSH are shown in Fig. 2. TRH increased both macro and free TSH within 30 minutes of administration (Fig. 2a). However, distinct circadian differences were observed between them (Fig. 2b), as low levels in the early morning were seen for macro TSH with increases throughout the day and into the evening, while free TSH levels were low in the daytime and then increased at night.

**Figure 3:**
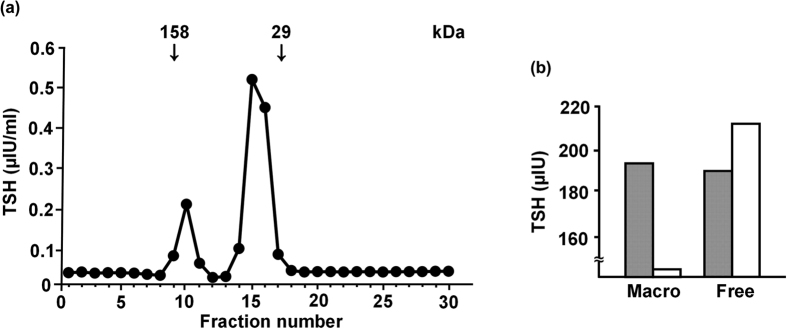
Gel filtration chromatography (**a**) and glycosylation analyses (**b**) of TSH. (**a**) Measurement of TSH in fractions using gel filtration chromatography. The main peak of TSH immunoreactivity was shown as a molecular mass of 44 kDa, while the other TSH peak fraction was found at 150 kDa. (**b**) Affinity for the Con A column by macro and free TSH. Open column, Con A-bound TSH; closed column, passed-through TSH; TSH, thyroid stimulating hormone.

To further analyze the structure of macro TSH, gel filtration chromatography and western blot analysis were performed using pooled serum samples from 10 patients. Gel filtration chromatography showed 2 peaks of TSH immunoreactivity corresponding to molecular masses of 44 and 150 kDa, confirming the presence of macro TSH (Fig. 3a). Marked smearing of TSHβ immunoreactivity was observed for the macro TSH fractions in the non-reduced condition (see online Supplementary Fig. 1a). In the native reduced condition, the majority of immunoreactivity of macro and free TSH was co-migrated, while a significant portion of macro TSH remained at the point of higher molecular mass. In both the denatured and reduced conditions, molecular sizes were indistinguishable between macro and free TSH. Moreover, after pretreatment of the fractions with PNGase, both the macro and free TSH fractions exhibited similar immunoreactive band motility (see online Supplementary Fig. 1b). Finally, the glycosylated structures of macro TSH were examined by analyzing binding affinity for the Con A column. When the macro TSH fraction was applied to the Con A column, all TSH immunoreactivity passed through the column, suggesting the presence of a structure of multi-branched N-linked glycans or O-glycanase. In sharp contrast, nearly half of the free TSH fraction bound to the Con A column, suggesting the presence of a structure composed of two-branched N-linked glycans (Fig. 3b).

## Discussion

The present study is the first to document that the level of macro TSH in serum is associated with sleep quality in patients with cardiovascular risk factors. This is also the first to demonstrate that changes in its glycosylated structure may be attributed to the macro TSH fraction in human serum.

Hypothyroidism and subclinical hypothyroidism have been suggested to be risk factors for sleep apnea syndrome^7^, and the macro TSH fraction has been detected in patients with subclinical hypothyroidism^10^. No previous study has examined the prevalence of macro TSH or its association with sleep disorders in patients without thyroid disease. Macro TSH is thought to be derived from the PT and can be markedly elevated (200–400 mIU/liter)^11^,^12^, potentially through associated autoimmune mechanisms^13^,^14^. Recently, it was reported that serum macro TSH in rodents was associated with seasonal physiology and behavior regulated by changes in day length^9^. In contrast to free TSH, macro TSH derived from the PT is under the control of melatonin signaling through the suprachiasmatic nucleus, which is known to regulate sleep behavior. In addition, PT-TSH was shown to be inert in regard to stimulation of the thyroid gland due to formation of a macro TSH complex and loss of bioactivity in the circulation. These results indicate the possibility that macro TSH is secreted from the PT and is under the control of sleep regulation (i.e., melatonin regulating) in humans, thus indicating its potential as a biological marker for sleep quality and rhythm.

To directly address this issue, we measured serum macro TSH and examined its relationship with quantitatively determined sleep quality. Among several sleep parameters measured by an actigraphy, parameters of either impaired sleep quality (increased sleep physical activity) or impaired sleep efficiency (decreased percent sleep) was significantly associated with high serum macro TSH in our cohort. Besides, the proportions of obese, dyslipidemic and diabetic were bigger in high macro TSH group. On the other hand, it is important to note that a higher serum level of macro TSH was significantly and independently associated with these poor sleep parameters even after adjustment for these potential confounding factors. In an analysis of all patients, levels of total TSH were different between the 2 groups with 5x higher total TSH in the macro TSH group, even though the difference is not statistically significant. Because of the higher variability in the macro TSH, it may be possible that hypothyroidism or at least a severe form of subclinical hypothyroidism may be included in the macro TSH group, which may be attributable to the association of macro TSH with poor sleep conditions^7^. To negate these possibilities, we further set subgroups of high and low macro TSH where total TSH levels were closely matched. Even in the analyses of the subgroups, high macro TSH was still significantly and independently associated with impaired sleep quality and less sleep efficiency with these relationships rather stronger as compared with the analysis of all subjects (Table 2). Thus, macro TSH level in serum appears to be a novel biomarker for sleep quality or efficiency in humans.

TSH synthesis in the anterior pituitary is generally stimulated by TRH and inhibited by thyroid hormone in a negative-feedback loop. TRH is under the control of the suprachiasmatic nucleus, which is fundamentally associated with circadian rhythm. In a TRH stimulation test, regulation of macro and free TSH is nearly identical, with the highest level at 30 minutes, followed thereafter by a gradual decrease. However, it remains unclear whether TRH itself is controlled by abnormal circadian rhythm, which may affect fluctuations of macro TSH that are apparently distinct from free TSH.

Recently, it was reported in rodents that pars distalis (PD)-TSH, free TSH, has sulfated biantennary N-glycans, while PT-TSH, macro TSH, has sialylated multibranched N-glycans in the circulation^9^. At present, the chemical structure of macro TSH has not been investigated in humans except in patients with very high serum TSH levels analyzed by gel filtration. In this study, we showed for the first time the presence of macro TSH in patients with normal serum TSH levels by polyethylene glycol precipitation, gel filtration, and poly-acrylamide gel electrophoresis under native and non-reduced conditions. In sharp contrast to the results in rodents^9^, under denatured, reduced or PNGase F-pretreated conditions, molecular sizes were indistinguishable between macro TSH and free TSH. However, we showed here in human that half of free TSH appears to be glycosylated by 2 branched N-linked glycans, while almost all macro TSH is glycosylated by multi-branched N-linked glycans as suggested by the affinities to lectin. Difference in glycosylation structure may be attributed to altered binding affinity to anti-TSH immunoglobulin to form a complex in serum.

The present study has several limitations. First, our results should not be generalized because of the small size of the cohort. In addition, due to its cross-sectional design, even though the relationships examined were explored in predictive terms, the study results cannot be used to show causal relationships between any of those factors.

In conclusion, an increase in serum macro TSH level was found to be associated with low sleep quality and regulated in a manner distinct from free TSH, possibly due to an altered glycosylation structure. Our findings highlight an important clinical point that macro TSH is present even in patients with a normal TSH level and is regulated by mechanisms that are different from those regulating TSH. Therefore, this point should be kept in mind when interpreting serum TSH measurements in regular clinical situations. Our findings add important information for understanding the pathophysiology of sleep conditions and open up new research aspects in the field of neuroendocrinology.

## Methods

### Study design and participants

Informed consent was obtained from all participants and the study was approved by the Ethics Committee of Hyogo College of Medicine (approved No. 948). The methods were carried out in accordance with approved guidelines.

This cross-sectional study included 314 patients not under treatment for hypo- or hyperthyroidism, who were registered in the HSCAA Study from October 2010 to July 2015. That study was conducted to examine the impact of sleep, autonomic imbalance, and subclinical atherosclerosis on cardiovascular events^5^. Patients with one or more cardiovascular risk factors, (obesity, smoking, presence of cardiovascular event history, hypertension, dyslipidemia, diabetes mellitus, chronic kidney disease), and being treated at the Division of Diabetes, Endocrinology and Metabolism, Hyogo Medical College Hospital (Hyogo, Japan) were analyzed. To completely negate the influence on analyses of the inclusion of patients with overt or subclinical hypothyroidism, we set subgroups of high and low macro TSH with total TSH levels were closely matched. In the subgroups, serum total TSH levels in high (n = 65) and low (n = 65) macro TSH groups were almost similar (p = 0.98).

### Assessment of classic cardiovascular risk factors

We obtained data regarding medical history, body mass index, and smoking status for each subject, and measured height and body weight. Previous cardiovascular events diagnosed by computed tomography or magnetic resonance imaging were defined as history of coronary heart disease (myocardial infarction or coronary intervention) or stroke (ischemic or hemorrhagic stroke). Hypertension was defined as systolic blood pressure ≥140 mmHg, diastolic blood pressure ≥90 mmHg, or treatment for hypertension. We defined dyslipidemia as the presence of low-density lipoprotein cholesterol ≥140 mg/dl, high density lipoprotein cholesterol ≤40 mg/dl, triglyceride level ≥150 mg/dl, or treatment for dyslipidemia^15^. Type 2 diabetes was diagnosed based on fasting plasma glucose ≥126 mg/dl, casual plasma glucose ≥200 mg/dl, 2 hour plasma glucose ≥200 mg/dl during a 75 g oral glucose tolerance test, or previous therapy for diabetes^16^. The estimated glomerular filtration rate (eGFR) in each patient was calculated using a new equation developed for Japanese subjects, as follows: eGFR (ml/min/1.73 m^2^) = 194 × age^−0.287^ × S-Cr^−1.094^ (if female, ×0.739)^17^.

### Determination of sleep conditions

An actigraph (Ambulatory Monitoring, Inc., Ardley, NY, USA) was used to examine sleep quality, as previously described^5^,^6^,^18^. This device converts signals produced from an acceleration sensor into samples collected at the present frequency in hertz. The samples are totalled over a user-specified time sampling interval called an “epoch”. Output from the actigraph is in the form of activity “counts”, which are recorded by converting acceleration units over a given epoch. The present subjects wore the accelerometer for 2 consecutive days. According to recommendations for clinical use of an actigraph^18^, sleep physical activity (mean activity counts per minutes by body motion during sleep), total sleep minutes (total minutes of sleep), percent sleep (percentage of time sleeping among time lying in bed), sleep efficiency (percentage of time scored as sleep), and time awake after sleep onset (number of minutes noted as awake during sleep time) were analyzed.

### TSH assays and polyethylene glycol precipitation

Blood specimens were collected regularly and centrifuged, then the supernatants were stored at −80 °C. Serum total TSH was measured using an electro-chemiluminescent assay (ECLIA) with an Elecsys 2010 (Roche Diagnostics K.K., Tokyo, Japan). Each sample was measured at cobas8000 (e602) according to the manufacturer’s protocol. Next, we used a polyethylene glycol (PEG) precipitation method to identify macroprolactin, as previously reported^19^. Equal volumes (200 μl) of a 25% solution of PEG and the serum sample were mixed and centrifuged at 5000 rpm for 5 minutes to precipitate γ-globulin fractions, then free TSH in the supernatant was measured. PEG-precipitated TSH (%) was calculated as follows: (total TSH-free TSH)/total TSH × 100, as previously described^10^.

### Gel filtration chromatography

To separate macro TSH from free TSH in the serum samples, gel filtration chromatography was performed using a Low/High Molecular Weight Kit (GE Healthcare, Japan). Pooled serum samples from 10 participants were passed through a 0.22 μm PES syringe filter (Starna Scientific, UK), then injected into a Superdex 200HR10/30 (GE Healthcare), which was equilibrated with 0.05 M of phosphate buffer in 0.15 M of NaCl solution adjusted to pH 7.0 and run at a flow-rate of 0.5 ml/minute on an AKTA Explorer 10 s (GE Healthcare). Chromatography was monitored using Prime View UNICON 5.31 software.

### Immunoprecipitation and western blotting

TSH was immunoprecipitated from the gel chromatography samples using a Pierce Crosslink Immunoprecipitation Kit (Thermo Fisher Scientific, USA) and an antibody against human TSHβ (Santa Cruz Sc-7813) (Santa Cruz Biotechnology, Dallas, TX, USA), according to the manufacturer’s protocol. To analyze TSH in a native condition, precipitated samples were released into 4 × sample buffer (0.25M Tris-HCL, pH 6.8, 40% glycerol, 0.02% bromophenol blue) (non-reducing) and 4 × sample buffer +20% 2-mercaptoethanol (reducing), then the samples (20 ul/well) were subjected to PAGE analysis (15%). To analyze TSH under a denaturing condition, precipitated samples were released by heating at 95 °C for 5 minutes in 4 × SDS (8%) -sample buffer, then samples (20 ul/well) were subjected to SDS-PAGE (10% or 12.5%). Under both the native and denaturing conditions, each sample was transferred to a PVDF membrane (Millipore, Japan) at 100 mA for 120 minutes using Trans-Blot SD (Bio-Rad, Japan). After blocking with 5% non-fat milk in 10 × TBST [1 M Tris-Hcl (pH 7.5) 100 ml, NaCl 43.83 g, H_2_O 500 ml, 0.05% Tween 20] for 60 minutes at room temperature (RT), blotted membranes were incubated with an anti-TSHβ antibody (Santa Cruz Sc-7813) at 1:200 dilution in TBST overnight at 4 °C. After washing the membranes 3 times in TBST buffer for 5 minutes at RT, they were incubated with a donkey antibody against goat IgG HRP (Sc-2020) at a 1:2000 dilution for 1 hour at RT. The membranes were then washed 3 times in TBST buffer for 5 minutes, and TSHβ bands were detected using an ECL Western Blotting Analysis System (GE Healthcare) or Super Signal West Pico Chemiluminescent Substrate (Thermo Fisher Scientific). To examine glycosylation of TSH, immunoprecipitated samples were treated with PNGase F (Peptide-N-Glycosidase F P0704S), which cleaves N-linked glycans, prior to performing western blotting analysis.

### Affinity for ConA column

To analyze whether the macro and free TSH fractions had distinct glycosylation structures, fractions obtained from gel filtration were applied to a HiTrap^®^ Con A 4B column (GE Healthcare), which can distinguish two-branched from multi-branched N-linked glycans or O-glycanase. Prior to application to the column, the samples were passed 3 times through an Amicon Ultra centrifugal filter (Millipore), then diluted with binding buffer (20 mM Tris-Hcl, 0.5 M NaCl, 1 mM MnCl_2_, 1 mM CaCl_2_ solution adjusted to pH 7.0) and passed through a 0.22 μm PES syringe filter (Starna Scientific), and finally injected into the Con A column. The pass-through fraction was collected first, then the column was washed 3 times with binding buffer and the adsorbed fraction was extracted 3 times with elution buffer (0.5 M methyl-α-D-glucopyranoside, 20 mM Tris-HCl, 0.5 M NaCl solution adjusted to pH 7.4). TSH levels in the Con A-negative and -positive fractions were measured using ECLIA.

### Statistical analysis

For statistical analysis, the patients were divided into low and high macro TSH groups, according to the median value for macro TSH (77.6%). Clinical characteristics and sleep parameters were compared between the groups, with multiple linear regression analysis used to explore the possibility of an independent relationship between a high level of macro TSH in serum and sleep quality. In addition, we investigated circadian changes in relation to serum macro and free TSH levels, and their changes following a TRH injection test. All statistical analyses were performed using the Statistical Package for Social Sciences software package (PASW Statistics version 18.0). All reported p values are 2-tailed and were considered to be statistically significant at <0.05.

## Additional Information

**How to cite this article**: Kadoya, M. *et al*. Serum Macro TSH Level is Associated with Sleep Quality in Patients with Cardiovascular Risks – HSCAA Study. *Sci. Rep.* **7**, 44387; doi: 10.1038/srep44387 (2017).

**Publisher's note:** Springer Nature remains neutral with regard to jurisdictional claims in published maps and institutional affiliations.

## Supplementary Material

Supplementary Information

## Figures and Tables

**Table 1:** Clinical characteristics of study participants.

Variables	All (n = 314)		P	Total TSH matched (n = 130)		
	Low macro TSH (n = 157)	High macro TSH (n = 157)		Low macro TSH (n = 65)	High macro TSH (n = 65)	P
Age, years	57.7 ± 13.3	59.8 ± 13.7	0.16	57.7 ± 1.6	60.2 ± 1.6	0.27
Male sex, n (%)	87 (55.4%)	91 (58.0%)	0.64	33 (50.8%)	38 (58.5%)	0.48
Body mass index, kg/m^2^	23.7 ± 4.9	25.2 ± 4.8	<0.01	23.0 ± 6.5	25.2 ± 0.5	<0.01
Current smoking, n (%)	41 (26.1%)	40 (25.5%)	0.89	15 (23.1%)	18 (27.7%)	0.68
CVD history, n (%)	25 (15.9%)	25 (15.9%)	1.00	10 (15.4%)	12 (18.5%)	0.81
Hypertension, n (%)	96 (61.1%)	107 (68.2%)	0.19	37 (56.9%)	47 (72.3%)	0.09
Dyslipidemia, n (%)	78 (49.7%)	110 (70.1%)	<0.01	31 (47.7%)	47 (72.3%)	<0.01
Diabetes mellitus, n (%)	49 (31.2%)	78 (49.7%)	<0.01	23 (35.4%)	35 (53.8%)	0.05
eGFR, ml/min/1.73 m^2^	80.8 ± 22.6	79.2 ± 21.8	0.53	82.8 ± 2.3	75.3 ± 2.8	0.04
Free T4 (ng/ml)	1.24 ± 0.21	1.22 ± 0.17	0.33	1.24 ± 0.01	1.22 ± 0.02	0.60
Total TSH (μIU/ml)	2.20 ± 1.40	10.76 ± 8.37	0.20	2.58 ± 0.14	2.58 ± 0.14	0.98
Free TSH (μIU/ml)	0.56 ± 0.35	0.71 ± 1.62	0.26	0.63 ± 0.03	0.48 ± 0.02	<0.01
Macro TSH (%)	73.8 ± 4.0	81.5 ± 3.7	NA	75.0 ± 0.2	81.1 ± 0.3	NA

Participants were divided into 2 groups according to the median value (77.6%) for macro TSH. In subgroup analysis total TSH levels were closely matched between the groups. Data are presented as the mean ± standard error and number (%) for dichotomous variables. P values are shown for comparisons of means between the groups (unrepeated t-test) or percentages (Chi-square test). CVD, cardiovascular disease history; eGFR, estimated glomerular filtration rate; TSH, thyroid-stimulating hormone; NA, not applicable.

**Table 2:** Multiple linear regression analyses of associations of macro TSH with sleep parameters.

Variables	Sleep physical activity		% sleep	
	β	P	β	P
All patients (n = 314)				
Macro TSH (high = 1, low = 0)	0.145	0.01	−0.150	<0.01
Adjusted R^2^	0.041	<0.01	0.047	<0.01
Total TSH matched patients (n = 130)				
Macro TSH (high = 1, low = 0)	0.288	<0.01	−0.263	<0.01
Adjusted R^2^	0.099	<0.01	0.098	<0.01

Multiple linear regression analyses were performed. The covariate in each model included age, male sex, body mass index, and presence of hypertension, dyslipidemia, or diabetes mellitus. TSH, thyroid-stimulating hormone; β, standard regression coefficient.
