# Supplementary material for: Serum Macro TSH Level is Associated with Sleep Quality in Patients with Cardiovascular Risks – HSCAA Study
Source: Sci Rep. 2017 Mar 13;7:44387. doi: 10.1038/srep44387 (PMC5346998; doi:10.1038/srep44387)
Supplement: Supplementary Information [file srep44387-s1.pdf]

Serum Macro TSH Level is Associated with Sleep Quality in Patients with Cardiovascular Risks – HSCAA Study

Manabu Kadoya[1], Sachie Koyama[1], Akiko Morimoto[1], Akio Miyoshi[1], Miki Kakutani[1], Kae Hamamoto[1], Masafumi Kurajoh[1], Takuhito Shoji[1], Yuji Moriwaki[1], Masahiro Koshiba[2], Tetsuya Yamamoto[1], Masaaki Inaba[3], Mitsuyoshi Namba[1], and Hidenori Koyama[1]*

[1]Department of Internal Medicine, Division of Diabetes, Endocrinology and Metabolism, Hyogo College of Medicine, 1-1 Mukogawa-cho, Nishinomiya, Hyogo 663-8501, Japan

[2]Department of Clinical Laboratory Medicine, Hyogo College of Medicine, 1-1 Mukogawa-cho, Nishinomiya, Hyogo 663-8501, Japan

[3]Department of Endocrinology, Metabolism and Molecular Medicine, Osaka City University Graduate School of Medicine, Osaka 545-8585, Japan

*Corresponding author:
Hidenori Koyama, M.D., Ph.D.
Department of Internal Medicine, Division of Diabetes, Endocrinology and Metabolism
Hyogo College of Medicine
1-1 Mukogawa-cho, Nishinomiya, Hyogo 663-8501, Japan
Tel: +81 798 45 6472, Fax: +81 798 45 6474
Email address: hkoyama@hyo-med.ac.jp

Supplementary Figure Legend

Supplementary Figure 1. Western blotting analyses of TSH.

(a) Western blotting analyses of macro and free TSH obtained from gel filtration were performed under native, non-reducing, and reducing conditions. Marked smearing of TSHβ immunoreactivity was observed for the macro TSH fractions in a non-reducing condition. (b) Macro and free TSH cleaved with PNGase F were analyzed by western blotting under denaturing and reducing condition. TSHβ was detected at the same molecular weight in both the macro and free TSH fractions.

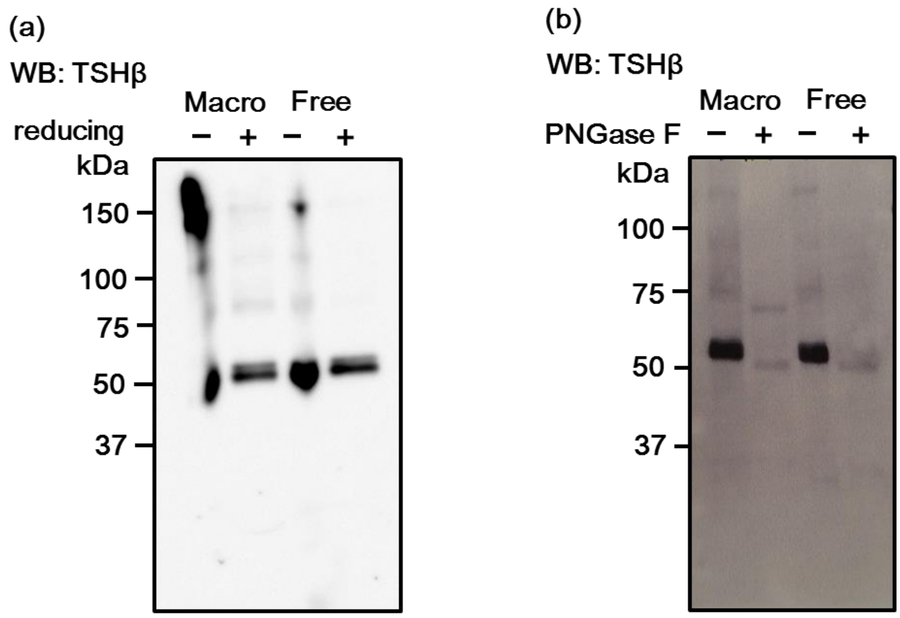

Supplementary Figure 1
